# Is hypoglycemia fear independently associated with health-related quality of life?

**DOI:** 10.1186/s12955-014-0167-3

**Published:** 2014-11-30

**Authors:** Lizheng Shi, Hui Shao, Yingnan Zhao, Nina A Thomas

**Affiliations:** Department of Global Health Systems and Development, Tulane University School of Public Health and Tropical Medicine, New Orleans, LA USA; College of Pharmacy, Xavier University of Louisiana, New Orleans, LA USA; US Health Services Research, Bristol-Myers Squibb, Princeton, NJ USA

**Keywords:** Antidiabetic agents, Health status, Hypoglycemia, Outcome assessment, Quality of life, Type 2 diabetes

## Abstract

**Objective:**

Patients may fear the symptoms and consequences associated with hypoglycemia. We tested whether fear of hypoglycemia is independently associated with poorer health-related quality of life (HRQOL).

**Research design and methods:**

Data were collected using direct-mail survey and enrollment information from adult commercial health plan enrollees with type 2 diabetes during a 12-month period (12/01/2008 to 11/30/2009). HRQOL was evaluated by the EuroQol (EQ)–5D index and 12-item Short Form Health Survey Mental Component Summary (SF-12 MCS) and Physical Component Summary (SF-12 PCS). Fear of hypoglycemia was assessed using the Hypoglycemia Fear Survey (HFS). Two ordinary least-squares (OLS) models of HRQOL controlling for demographics and illness characteristics were specified, and OLS regression coefficients and statistical inferences were compared. Model 1 included 1 variable of hypoglycemia symptoms; Model 2 included both hypoglycemia symptoms and HFS score.

**Results:**

Of 3999 patients contacted, 813 responded to the survey. Model 1: hypoglycemia symptoms alone were associated with worse HRQOL (SF-12 MCS and SF-12 PCS scores and EQ-5D utility score; all *P* < 0.05). Model 2: hypoglycemia symptoms were significantly associated only with SF-12 MCS score. HFS total score was significantly associated with all 3 HRQOL scores. Hypoglycemia symptoms, Hispanic ethnicity, and longer diabetes duration were associated with greater hypoglycemia fear. Higher income, white race, and treatment without sulfonylurea or insulin were associated with less hypoglycemia fear (all *P* < 0.05).

**Conclusions:**

In addition to the effect of symptomatic hypoglycemia on HRQOL, fear of hypoglycemia was independently associated with lower overall health status and mental and physical health.

**Electronic supplementary material:**

The online version of this article (doi:10.1186/s12955-014-0167-3) contains supplementary material, which is available to authorized users.

Hypoglycemia is associated with increased risk of a variety of adverse clinical outcomes in patients with type 2 diabetes, including microvascular events (eg, nephropathy, retinopathy, neuropathy), macrovascular events (eg, heart failure, peripheral vascular disease, myocardial events, stroke), and death [1-3]. High rates of hypoglycemia symptoms (38%–63%) [4-6] and hypoglycemia episodes (14%–23%; based on low blood sugar) [7,8] are observed in patients with type 2 diabetes in general clinical practice. Hypoglycemia is primarily associated with treatment with insulin and insulin secretagogues [9]. A nationwide cohort study demonstrated that patients who experience hypoglycemia are more likely to be using insulin and sulfonylureas than those who do not experience hypoglycemia. Hypoglycemia, use of insulin, and use of sulfonylureas are associated with increased risk for major cardiovascular (CV) events, CV disease, and death [3]. Risk of hypoglycemia also increases with age and duration of diabetes [10].

Patients may fear the symptoms associated with a mild episode of hypoglycemia as well as the consequences of severe hypoglycemia, which may include seizure or loss of consciousness [11].

Fear of hypoglycemia is a considerable problem in patients with diabetes [5,12] and may itself represent a barrier to glycemic control. Hypoglycemia is associated with lower health-related quality of life (HRQOL) [5,7,8,13], depression [7], and lower treatment satisfaction [4,5], which could undermine treatment adherence [7]. Fear of hypoglycemia may lead to preventive coping behaviors, such as taking less insulin or increasing food intake [12,14], that negatively affect glycemic control. Fear of hypoglycemia may also lead physicians to limit the aggressiveness of therapy [15].

In a cross-sectional survey study, we found that symptomatic hypoglycemia was associated with worse HRQOL as well as greater fear of hypoglycemia [16]. Compared with patients who did not report a history of symptomatic hypoglycemia, patients with a confirmed history of symptomatic hypoglycemia had higher scores on the Hypoglycemia Fear Survey (HFS) Behavior and Worry subscales (indicating more fear of and worry about hypoglycemia; each *P* < 0.001), lower scores on the EuroQol (EQ)–5D index (indicating poorer health status, *P* < 0.001), and lower scores on the 12-item Short Form Health Survey (SF-12) Mental Component Summary (MCS) score (*P* < 0.001) and Physical Component Summary (PCS) score (*P* = 0.002). The objective of this retrospective analysis was to examine the relationships among symptomatic hypoglycemia, patient self-reported fear of hypoglycemia, and HRQOL while controlling for patient baseline demographic and disease characteristics. We tested the hypothesis that, beyond the effect of symptomatic hypoglycemia itself on patient HRQOL, fear of hypoglycemia is independently associated with lower HRQOL.

## Research design and methods

### Study design

Detailed methodology for this cross-sectional observational research study was previously published [16]. The study combined patient-reported data collected through mail surveys and retrospective analysis of administrative claims data (medical data, pharmacy data, and enrollment information) from a large US health plan (the Life Sciences Research Database). The study protocol was approved by a central institutional review board (IRB) prior to study initiation. And due to the fact that the patients in our study were identified from administrative claims data, the study concept was also approved by the large US health plan prior to commencement of the study. Participants were adult enrollees in the health plan with a diagnosis of type 2 diabetes and ≥2 pharmacy claims for an oral antidiabetic medication (OAD) with or without insulin identified in the administrative claims data from December 1, 2008, to November 30, 2009.

### Patient survey

For the survey, patients (N = 3999) were contacted directly by mail. The patient survey assessed demographic variables, age at time of type 2 diabetes diagnosis, family history of diabetes, and smoking status. Patients were asked about their current OAD, duration of treatment, and modifications to their treatment within the previous 6 months. Medication adherence was assessed using the Morisky self-report medication adherence 4-item questionnaire [17].

Fear of hypoglycemia was evaluated using the HFS [18]. This validated assessment lists 15 behaviors that patients may engage in to avoid low blood sugar, with potential consequences, and 18 items related to low blood sugar about which patients with diabetes may be concerned. The recall period was 6 months. A total score, Behavior subscale score (sum of the 15 behavioral items), and Worry subscale score (sum of the 18 worry items) were generated. In addition, patients were asked whether they had changed their medication or were afraid to change medications because of hypoglycemia.

Health-related quality of life was assessed using 2 instruments. Overall health status was assessed using the EQ-5D, a standardized measure that rates 5 dimensions of health (mobility, self-care, usual activities, pain/discomfort, anxiety/depression) across 3 levels (no problems, some/moderate problems, and extreme problems) [19,20]. The index value (ie, health utility) between 0 (death) and 1 (perfect health) was calculated using the value sets (weights) from a US population sample. The other instrument was the SF-12 [21], which can produce an MCS and a PCS. Summary scores range from 0 to 100, with a higher score indicating a better quality of life in mental and physical components. Hypoglycemia was defined based on American Diabetes Association symptoms [22] and blood glucose readings at the time that symptoms occurred.

The survey packet we used in this study contained a pre-paid cash incentive of $10 to the patients, along with the invitation letter printed on letterhead, the IRB-approved consent form, the survey instrument printed in booklet form, and a postage-paid business reply envelope. We sent reminders to the patients 2 and 4 weeks following the initial survey to increase our response rate.

### Statistical analyses

Survey variables (eg, demographics and clinical characteristics) were analyzed descriptively, and bivariate comparisons of demographic characteristics and outcome measures were performed using appropriate tests (eg, *t*-test or chi-square test) based on the distribution of the measure. Multivariate analyses of HFS, SF-12, and EQ-5D outcomes were conducted using ordinary least squares. Specific predictors (ie, explanatory variables) to be included were selected primarily based on clinical rationale. Associations among symptomatic hypoglycemia, fear of hypoglycemia, and HRQOL were modeled in 2 ordinary least-squares regressions, controlling for sociodemographics, illness characteristics, and treatment factors [16]. Model 1 of the HRQOL = f (symptomatic hypoglycemia) tested the associations between symptomatic hypoglycemia and HRQOL, and based on previously published results [16], these were expected to be statistically significant. Model 2, which was the primary assessment of the study hypothesis, added 1 additional variable of hypoglycemia fear and tested the impact that fear of hypoglycemia had on health status independently of the impact of hypoglycemia symptoms. Following standard procedure, regression diagnostics were performed to assess goodness of fit and violations of model assumptions (eg, multicollinearity, heteroskedasticity) for each model. Any violations of the model were noted and appropriate corrections made to the data. The fitted and observed data were examined to uncover outliers, their effect on the analysis, and possible misspecification of the initial equation. SAS 9.2 software (SAS Institute, Inc., Cary, NC) was used for statistical analyses [23]. The significance level was set at 0.05 (2-tailed).

Because different classes of antidiabetic drugs may be more likely to cause hypoglycemia [24-27], the association between fear for hypoglycemia and quality of life might also be different across different patient subgroups of antidiabetic medication users. Due to this concern, the same statistical analysis procedures were conducted on two patient subgroups of antidiabetic medication users: insulin users and sulfonylurea users.

## Results

### Patient survey results

Of 3999 patients contacted, 813 responded with a complete survey (response rate, 20.3%). Of these, 578 (71.1%) reported ≥1 hypoglycemia symptom within the previous 3 months. Among the population characteristics assessed, only OAD group and sex showed a statistically significant difference between patients with and without symptomatic hypoglycemia (Table 1). Patients in the OAD plus insulin group were more likely to report ≥1 hypoglycemia symptom (83.3% [244/293]) compared with those in the sulfonylurea without insulin group (69.9% [172/246]) or the nonsulfonylurea OAD without insulin group (59.1% [162/274]; *P* < 0.001). Significantly more women (77.1% [263/341]) than men (66.7% [315/472]) reported ≥1 hypoglycemia symptom.

**Table 1:** **Population characteristics between patients who reported versus those who did not report symptoms of hypoglycemia**

**Characteristic**, **n (%)**	**No hypoglycemia** **(n = 235)**	**Hypoglycemia** **(n = 578)**	***P*** value*
Age, y			
<65	203 (86.4)	525 (90.8)	0.0603
≥65	32 (13.6)	53 (9.2)	
Sex			
Female	78 (33.2)	263 (45.5)	0.0013
Male	157 (66.8)	315 (54.5)	
Family history of diabetes			
No	58 (26.9)	110 (20.4)	0.0528
Yes	158 (73.2)	430 (79.6)	
Treatment			
Any OAD with insulin	49 (20.9)	244 (42.2)	
SU without insulin	74 (31.5)	172 (29.8)	<.0001
Non-SU OAD without insulin	112 (47.7)	162 (28.0)	
Diabetes treatment period, y			
<1	45 (19.2)	96 (16.7)	0.4765
1–2	70 (29.9)	151 (26.3)	
3–5	63 (26.9)	167 (29.1)	
6–10	36 (15.4)	91 (15.9)	
>10	20 (8.6)	69 (12.0)	
Ethnicity			
White			
No	52 (22.5)	161 (28.3)	0.0961
Yes	179 (77.5)	409 (71.8)	
Black or African American			
No	196 (84.9)	469 (82.3)	0.3806
Yes	35 (15.2)	101 (17.7)	
Hispanic or Latino			
No	209 (89.7)	525 (92.3)	0.2360
Yes	24 (10.3)	44 (7.7)	
Marital status			
Single, never married	25 (10.7)	68 (11.8)	0.6079
Married	167 (71.4)	390 (67.7)	
Divorced	32 (13.7)	82 (14.2)	
Separated	2 (0.9)	14 (2.4)	
Widowed	8 (3.4)	22 (3.8)	
Annual household income, $US			
<25,000	13 (5.9)	44 (8.0)	0.7025
25,000–49,999	68 (30.6)	169 (30.8)	
50,000–74,999	42 (18.9)	116 (21.1)	
75,000–99,999	36 (16.2)	78 (14.2)	
>100,000	63 (28.4)	142 (25.9)	
Education			
Less than secondary	9 (3.9)	14 (2.5)	0.8425
Some secondary	4 (1.7)	14 (2.5)	
Secondary or equivalent	53 (22.8)	131 (22.9)	
Tertiary but no degree	60 (25.8)	166 (29.0)	
2-year undergraduate degree	28 (12.0)	59 (10.3)	
>2-year undergraduate degree	51 (21.9)	199 (20.8)	
Graduate school	28 (12.0)	69 (12.1)	
US Region			
Northeast	13 (5.5)	33 (5.7)	0.2144
Midwest	29 (12.3)	54 (9.3)	
South	181 (77.0)	440 (76.1)	
West	12 (5.1)	51 (8.8)	
Morisky adherence			
Low	123 (52.8)	265 (46.3)	0.3058
Relatively low	71 (30.5)	186 (32.5)	
Middle	28 (12.0)	77 (13.4)	
Relatively high	10 (4.3)	36 (6.3)	
High	1 (0.4)	9 (1.6)	

*OAD* = oral antidiabetic medication; *SU* = sulfonylurea.

*Chi-square test.

**Figure 1:**
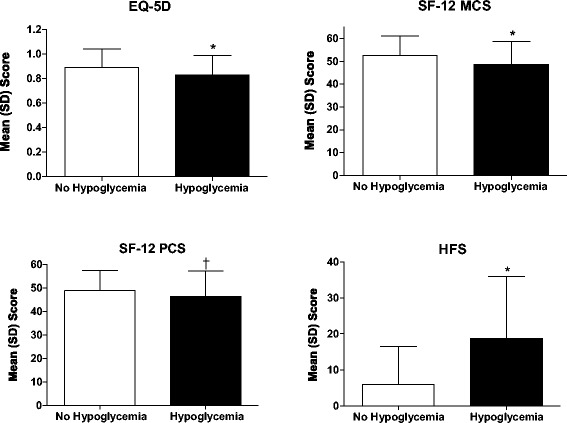
**Comparison of health**-**related quality**-**of**-**life scores and fear of hypoglycemia between patients who reported** **(n = 235)** **vs those who did not report** **(n = 578)** **symptoms of hypoglycemia.** EQ-5D=EuroQol-5D scale; HFS = Hypoglycemia Fear Survey; MCS = Mental Component Summary; PCS = Physical Component Summary; SF-12 = 12-item Short Form Health Survey. *P < 0.0001, ^†^ *P* = 0.0007 by *t* test.

Symptomatic hypoglycemia was associated with more fear of hypoglycemia (higher total HFS score) and worse HRQOL (lower SF-12 MCS, SF-12 PCS, and EQ-5D scores; each, *P* < 0.05; Figure 1). Table 2 presents the associations between the HFS and various factors. The presence of symptomatic hypoglycemia was associated with greater fear of hypoglycemia and had the strongest association (regression coefficient >10 points of HFS total score, *P* < 0.0001). Other factors also showed statistically significant associations (Table 2). Higher income was associated with less fear of hypoglycemia (*P* < 0.0001), as was white racial categorization compared with nonwhite racial categorizations (*P* < 0.0001). Hispanic ethnicity was associated with greater fear of hypoglycemia (*P* = 0.03). Longer duration with a diagnosis of diabetes and better self-reported adherence were also associated with greater fear of hypoglycemia (each *P* < 0.0001).

**Table 2:** **Influencing factors of hypoglycemia fear survey total scores**

**Variable (reference)**	**Regression coefficient**	**Standard error**	***P*** **value**	**95%** **CI**	
				**Lower**	**Upper**
Hypoglycemia	10.47	1.28	<0.0001	7.95	12.99
Age (<65 y)					
≥65 y	−1.23	1.91	0.52	−4.98	2.51
Sex (male)					
Female	−1.76	1.23	0.15	−4.17	0.66
Body mass index	−0.10	0.08	0.22	−0.25	0.06
Family history of diabetes	−0.58	1.37	0.67	−3.27	2.10
Treatment (SU without insulin)					
Non-SU OAD without insulin	−2.25	1.44	0.12	−5.09	0.58
Any OAD with insulin	2.09	1.42	0.14	−0.71	4.89
Duration of current diabetes medication	−0.70	0.47	0.14	−1.63	0.23
Ethnicity					
White (not white)	−4.56	1.35	<0.0001	−7.22	−1.91
Hispanic or Latino (not Hispanic or Latino)	4.74	2.13	0.03	0.56	8.92
Married (currently not married)	−0.04	1.29	0.97	−2.59	2.50
Household income level	−1.94	0.47	<0.0001	−2.86	−1.03
Education (less than secondary)	0.75	3.56	0.83	−6.25	7.75
Region (Northeast)					
Midwest	1.24	2.82	0.66	−4.29	6.77
South	1.41	2.33	0.55	−3.17	5.99
West	0.21	3.14	0.95	−5.96	6.37
Diabetes duration (y)	0.36	0.08	<0.0001	0.20	0.52
Morisky adherence	1.72	0.60	<0.0001	0.54	2.91

*OAD* = oral antidiabetic medication; *SU* = sulfonylurea.

The ordinary least-squares model of HRQOL using symptomatic hypoglycemia only and controlling for demographics and illness characteristics (Model 1, Table 3) yielded regression coefficients for symptomatic hypoglycemia on HRQOL scores. The associations between symptomatic hypoglycemia and EQ-5D score and SF-12 MCS score were statistically significant (each *P* < 0.05). In Model 2, which included the additional variable of HFS, the HFS score was significantly associated with scores on all 3 HRQOL outcomes (each *P* < 0.0001), whereas symptomatic hypoglycemia showed a statistically significant association only for the SF-12 MCS (*P* = 0.022). The fully specified regression models for Table 3 are provided in Additional file 1: Tables S1.1-S1.6.

**Table 3:** **Role of hypoglycemia fear on health**-**related quality of life using alternative models**

**Variable**	**EQ**-**5D (US)**			**SF**-**12 MCS**			**SF**-**12 PCS**		
	**β**	***P*** **value**	**95%** **CI for β**	**β**	***P*** **value**	**95%** **CI for β**	**β**	***P*** **value**	**95%** **CI for β**
Model 1									
Hypo	0.041	0.002	−0.067 to −0.015	−3.628	<0.0001	−5.259 to −1.998	−0.974	0.247	−2.623 to 0.675
Model 2									
Hypo	−0.014	0.303	−0.040 to 0.013	−1.937	0.022	−3.591 to −0.283	0.579	0.500	−1.104 to 2.262
HFS	−0.003	<0.0001	−0.003 to −0.002	−0.162	<0.0001	−0.210 to −0.115	−0.149	<0.0001	−0.197 to −0.101

β = regression coefficient; *EQ* = EuroQol; Hypo = symptomatic hypoglycemia; *HFS* = Hypoglycemia Fear Survey; *MCS* = Mental Component Summary; *PCS* = Physical Component Summary; *SF*-12 = 12-item Short Form Health Survey.

Subgroup analyses were conducted for insulin users (Table 4) and sulfonylurea users (Table 5). For insulin users (n = 293), Model 1 showed that the symptomatic hypoglycemia was not associated with 3 HRQOL scores (each *P* > 0.05). Model 2 showed that the HFS score was still significantly associated with scores on all 3 HRQOL outcomes (each *P* < 0.05), whereas symptomatic hypoglycemia showed no statistically significant association with any of the 3 HRQOL outcomes (each *P* > 0.05). For sulfonylurea users (n = 246), Model 1 showed that the symptomatic hypoglycemia was associated with EQ-5D scores and SF-12 MCS scores (each *P* < 0.05). Model 2 showed that the HFS score was significantly associated with scores on all 3 HRQOL outcomes (each *P* < 0.05), whereas symptomatic hypoglycemia was only significantly associated with EQ-5D scores and SF-12 MCS scores (each *P* < 0.05). The fully specified regression models for Table 4 and Table 5 are provided in Additional file 1: Tables S2.0-S3.5.

**Table 4:** **Role of hypoglycemia fear on health**-**related quality of life using alternative models** (**Insulin Subgroup**)

**Variable**	**EQ**-**5D** **(US)**			**SF**-**12 MCS**			**SF**-**12 PCS**		
	**β**	***P*** **value**	**95%** **CI for β**	**β**	***P*** **value**	**95%** **CI for β**	**β**	***P*** **value**	**95%** **CI for β**
Model 1									
Hypo	−0.001	0.96	−0.058 to 0.055	−2.506	0.137	−5.813 to 0.801	−1.668	0.359	−5.244 to 1.908
Model 2									
Hypo	0.029	0.311	−0.027 to 0.086	−0.285	0.863	−3.534 to 2.965	−0.358	0.847	−4.011 to 3.296
HFS	−0.003	<0.001	−0.004 to −0.001	−0.188	<0.0001	−0.259 to −0.116	−0.111	<0.007	−0.191 to −0.031

β = regression coefficient; *EQ* = EuroQol; Hypo = symptomatic hypoglycemia; *HFS* = Hypoglycemia Fear Survey; *MCS* = Mental Component Summary; *PCS* = Physical Component Summary; SF-12 = 12-item Short Form Health Survey.

**Table 5:** **Role of hypoglycemia fear on health**-**related quality of life using alternative models** (**Sulfonylurea Subgroup**)

**Variable**	**EQ**-**5D** **(US)**			**SF**-**12 MCS**			**SF**-**12 PCS**		
	**β**	***P*** **value**	**95%** **CI for β**	**β**	***P*** **value**	**95%** **CI for β**	**β**	***P*** **value**	**95%** **CI for β**
Model 1									
Hypo	−0.077	0.001	−0.123 to −0.031	−6.531	<0.001	−9.606 to −3.456	−1.815	0.241	−4.857 to 1.226
Model 2									
Hypo	−0.060	0.014	−0.107 to -0.013	−5.300	0.001	−8.453 to −2.147	0.056	0.971	−2.973 to 3.084
HFS	−0.002	0.014	−0.004 to −0.000	−0.140	0.008	−0.243 to −0.038	−0.213	<0.001	−0.311 to −0.114

β = regression coefficient; *EQ* = EuroQol; Hypo = symptomatic hypoglycemia; *HFS* = Hypoglycemia Fear Survey; *MCS* = Mental Component Summary; *PCS* = Physical Component Summary; SF-12 = 12-item Short Form Health Survey.

## Discussion

In patients with type 2 diabetes treated with antihyperglycemic therapy, hypoglycemia imposes a significant physical and psychological burden [28]. Previously, we demonstrated that hypoglycemia is significantly associated with poorer HRQOL as well as increased fear of hypoglycemia [16]. Other studies have shown an association between the severity of hypoglycemia and increased fear of hypoglycemia [29-31]. This retrospective analysis was conducted to test the hypothesis that fear of hypoglycemia is itself independently associated with poorer HRQOL. Results support this hypothesis, demonstrating a significant independent association between fear of hypoglycemia and scores on the EQ-5D and SF-12 PCS and MCS. This relationship was demonstrated for both insulin and sulfonylurea users. These results are consistent with previous cross sectional studies that have shown an association between fear of hypoglycemia and HRQOL [29,32].

A significant association was observed between symptomatic hypoglycemia and EQ-5D and SF-12 MCS scores in Model 1, whereas only the association with SF-12 MCS was significant after incorporating the HFS into the model. In contrast, fear of hypoglycemia demonstrated a significant association with all 3 HRQOL outcomes. This finding suggests that, statistically, fear of hypoglycemia may be a more important predictor than hypoglycemia itself of patient well-being and health status.

Multiple factors associated with fear of hypoglycemia were identified. Consistent with our study [16] and other reports [29,31,33], symptomatic hypoglycemia had the strongest (positive) association with fear of hypoglycemia. Thus, it appears that in patients with type 2 diabetes, experiencing an event of hypoglycemia increases rather than diminishes fear of such an event. An important clinical practice implication of this finding is the need for healthcare professionals to be cognizant of the fear of hypoglycemia and take steps to address this fear and related behaviors.

In the current analysis, Hispanic/Latino ethnicity, duration of diabetes, and self-reported adherence also showed a positive association with fear of hypoglycemia, whereas household income and white race showed a negative association. These data suggest that demographic variables might be helpful in predicting those patients most likely to experience fear of hypoglycemia. The positive association between duration of diabetes and fear of hypoglycemia could reflect greater insulin use in patients with a longer duration of disease. Alternatively, an independent association between these variables would seem to further indicate that experiencing hypoglycemia increases fear of the event.

As no causality can be inferred in this cross-sectional analysis, the positive relationship between self-reported adherence and fear of hypoglycemia is difficult to interpret. In a previous study, patients reporting hypoglycemia symptoms were more likely to report barriers to adherence (eg, bothered by medication side effects, unable to follow plans) [4]. In addition, data suggest that patients who have experienced a hypoglycemic episode are likely to initiate preventive behaviors that may include modification of their dosing regimen [14,16]. Although these findings may seem at odds with one another, it is reasonable to expect that patients adherent to strict glycemic control regimens would have more reason to be fearful of hypoglycemia. Additional studies with longitudinal data collection are needed to fully understand the variables that predict fear of hypoglycemia.

Predictors of hypoglycemia in patients with type 2 diabetes are better understood. In the current analysis, more women versus men and more patients treated with OAD plus insulin versus sulfonylurea without insulin or nonsulfonylurea OAD without insulin reported hypoglycemia. The difference in hypoglycemia with respect to diabetes therapy is to be expected, given that insulin and insulin secretagogues are the most common cause of hypoglycemia in patients with type 2 diabetes [15]. The observation that more women than men reported hypoglycemic symptoms was unexpected but has been observed in another study [29] and could possibly reflect sex-related differences in self-reporting of hypoglycemic symptoms.

This analysis has some limitations. Because the study was cross-sectional, no causal relationships can be inferred from the findings. Data on the frequency and severity of hypoglycemic episodes were not collected so their contribution to fear of hypoglycemia could not be assessed. Also, because the survey design used a convenience sample of patients that included only those who were members of specific health plans, with voluntary participation, survey participants may not be representative of all patients with type 2 diabetes. The study also relied on patient self-report, and the survey had a patient recall period of 6 months. Longitudinal cohort studies are warranted to further understand the impact of hypoglycemia and to test potential interventions to address the issue of hypoglycemia fear to improve health for patients with diabetes. In conclusion, in addition to symptomatic hypoglycemia itself, fear of hypoglycemia was independently associated with lower HRQOL (overall health status, mental health, and physical health) in patients with type 2 diabetes. There is an unmet need for patient education programs that address patient fear of hypoglycemia and use of medications with a lower risk of hypoglycemia.

## Additional file

Additional file 1:
**Table S1.1** Fully Specified Hypoglycemia Model 1: EQ-5D (US). **Table S1.2** Fully Specified Hypoglycemia Model 1: SF-12 MCS. **Table S1.3** Fully Specified Hypoglycemia Model 1: SF-12 PCS. **Table S1.4** Fully Specified Hypoglycemia Model 2: EQ-5D (US). **Table S1.5** Fully Specified Hypoglycemia Model 2: SF-12 MCS. **Table S1.6** Fully Specified Hypoglycemia Model 2: SF-12 PCS. **Table S2.0** Hypoglycemia Model 1: Insulin Subgroup Measured by EQ-5D(US). **Table S2.1** Hypoglycemia Model 1: Insulin Subgroup Measured by SF-12 MCS. **Table S2.2** Hypoglycemia Model 1: Insulin Subgroup Measured by SF-12 PCS. **Table S2.3** Hypoglycemia Model 2: Insulin Subgroup Measured by EQ-5D(US). **Table S2.4** Hypoglycemia Model 2: Insulin Subgroup Measured by SF-12 MCS. **Table S2.5** Hypoglycemia Model 2: Insulin Subgroup Measured by SF-12 PCS. **Table S3.0** Hypoglycemia Model 1: Sulfonylurea Subgroup Measured by EQ-5D (US). **Table S3.1** Hypoglycemia Model 1: Sulfonylurea Subgroup Measured by SF-12 MCS. **Table S3.2** Hypoglycemia Model 1: Sulfonylurea Subgroup Measured by SF-12 PCS. **Table S3.3** Hypoglycemia Model 2: Sulfonylurea Subgroup Measured by EQ-5D (US). **Table S3.4** Hypoglycemia Model 2: Sulfonylurea Subgroup Measured by SF-12 MCS. **Table S3.5** Hypoglycemia Model 2: Sulfonylurea Subgroup Measured by SF-12 PCS.
