# Supplementary material for: Is hypoglycemia fear independently associated with health-related quality of life?
Source: Health Qual Life Outcomes. 2014 Nov 30;12:167. doi: 10.1186/s12955-014-0167-3 (PMC4268814; doi:10.1186/s12955-014-0167-3)
Supplement: Additional file 1: — Table S1.1 Fully Specified Hypoglycemia Model 1: EQ-5D (US). Table S1.2 Fully Specified Hypoglycemia Model 1: SF-12 MCS. Table S1.3 Fully Specified Hypoglycemia Model 1: SF-12 PCS. Table S1.4 Fully Specified Hypoglycemia Model 2: EQ-5D (US). Table S1.5 Fully Specified Hypoglycemia Model 2: SF-12 MCS. Table S1.6 Fully Specified Hypoglycemia Model 2: SF-12 PCS. Table S2.0 Hypoglycemia Model 1: Insulin Subgroup Measured by EQ-5D(US). Table S2.1 Hypoglycemia Model 1: Insulin Subgroup Measured by SF-12 MCS. Table S2.2 Hypoglycemia Model 1: Insulin Subgroup Measured by SF-12 PCS. Table S2.3 Hypoglycemia Model 2: Insulin Subgroup Measured by EQ-5D(US). Table S2.4 Hypoglycemia Model 2: Insulin Subgroup Measured by SF-12 MCS. Table S2.5 Hypoglycemia Model 2: Insulin Subgroup Measured by SF-12 PCS. Table S3.0 Hypoglycemia Model 1: Sulfonylurea Subgroup Measured by EQ-5D (US). Table S3.1 Hypoglycemia Model 1: Sulfonylurea Subgroup Measured by SF-12 MCS. Table S3.2 Hypoglycemia Model 1: Sulfonylurea Subgroup Measured by SF-12 PCS. Table S3.3 Hypoglycemia Model 2: Sulfonylurea Subgroup Measured by EQ-5D (US). Table S3.4 Hypoglycemia Model 2: Sulfonylurea Subgroup Measured by SF-12 MCS. Table S3.5 Hypoglycemia Model 2: Sulfonylurea Subgroup Measured by SF-12 PCS. [file 12955_2014_167_MOESM1_ESM.docx]

Additional file 1

Table S1.1 Fully Specified Hypoglycemia Model 1: EQ-5D (US)

| Variable (reference) | Regression Coefficient | Standard Error | P Value | 95% CI | |
| --- | --- | --- | --- | --- | --- |
|  |  |  |  | Lower | Upper |
| Hypoglycemia | −0.041 | 0.013 | 0.002 | −0.067 | −0.015 |
| Age >65 years | 0.018 | 0.020 | 0.353 | −0.020 | 0.057 |
| Sex (male) | 0.019 | 0.013 | 0.140 | −0.006 | 0.044 |
| DM family history | 0.001 | 0.014 | 0.918 | −0.026 | 0.029 |
| Cohort: SU without insulin |  |  |  |  |  |
| AD with insulin | −0.013 | 0.015 | 0.397 | −0.042 | 0.017 |
| Non-SU AD without insulin | 0.002 | 0.015 | 0.909 | −0.028 | 0.031 |
| BMI | −0.003 | 0.001 | 0.001 | −0.005 | −0.002 |
| Duration of current AD medication | 0.001 | 0.005 | 0.764 | −0.008 | 0.011 |
| White | −0.009 | 0.014 | 0.505 | −0.037 | 0.018 |
| Hispanic or Latino | 0.004 | 0.022 | 0.864 | −0.039 | 0.047 |
| Marital status (currently not married) | −0.012 | 0.013 | 0.367 | −0.039 | 0.014 |
| Household income level | 0.016 | 0.005 | 0.001 | 0.006 | 0.025 |
| Education (lower than high school) | 0.094 | 0.037 | 0.011 | 0.022 | 0.165 |
| Region (Northeast) |  |  |  |  |  |
| Midwest | −0.016 | 0.029 | 0.571 | −0.073 | 0.040 |
| South | −0.015 | 0.024 | 0.525 | −0.062 | 0.032 |
| West | 0.004 | 0.033 | 0.896 | −0.060 | 0.068 |
| Diabetes duration (years) | −0.003 | 0.001 | 0.001 | −0.004 | −0.001 |
| Morisky adherence | −0.011 | 0.006 | 0.091 | −0.023 | 0.002 |

AD = antidiabetic drug; BMI = body mass index; DM = diabetes mellitus; EQ-5D = EuroQol-5D index; SU = sulfonylurea.

Table S1 2 Fully Specified Hypoglycemia Model 1: SF-12 MCS

| Variable (reference) | Regression Coefficient | Standard Error | P Value | 95% CI | |
| --- | --- | --- | --- | --- | --- |
|  |  |  |  | Lower | Upper |
| Hypoglycemia | −3.628 | 0.830 | <.0001 | −5.259 | −1.998 |
| Age >65 years | 2.361 | 1.231 | 0.056 | −0.056 | 4.777 |
| Sex (male) | 1.803 | 0.794 | 0.024 | 0.243 | 3.363 |
| DM family history | −0.304 | 0.879 | 0.730 | −2.029 | 1.421 |
| Cohort: SU without insulin |  |  |  |  |  |
| AD with insulin | −0.631 | 0.924 | 0.495 | −2.445 | 1.183 |
| Non-SU AD without insulin | −0.746 | 0.933 | 0.425 | −2.578 | 1.087 |
| BMI | −0.070 | 0.051 | 0.166 | −0.170 | 0.029 |
| Duration of current AD medication | 0.296 | 0.306 | 0.333 | −0.304 | 0.897 |
| White | −0.787 | 0.877 | 0.370 | −2.509 | 0.935 |
| Hispanic or Latino | −2.044 | 1.368 | 0.136 | −4.731 | 0.642 |
| Marital status (currently not married) | 0.706 | 0.838 | 0.400 | −0.941 | 2.352 |
| Household income level | 0.274 | 0.303 | 0.366 | −0.320 | 0.868 |
| Education (lower than high school) | 4.987 | 2.286 | 0.030 | 0.498 | 9.476 |
| Region (Northeast) |  |  |  |  |  |
| Midwest | 1.084 | 1.809 | 0.549 | −2.467 | 4.636 |
| South | 0.263 | 1.497 | 0.861 | −2.677 | 3.204 |
| West | 2.699 | 2.035 | 0.185 | −1.298 | 6.696 |
| Diabetes duration (years) | −0.017 | 0.052 | 0.749 | −0.120 | 0.086 |
| Morisky adherence | −2.188 | 0.388 | <.0001 | −2.951 | −1.425 |

AD = antidiabetic drug; BMI = body mass index; DM = diabetes mellitus; SF-12 MCS = 12-item Short Form Health Survey Mental Component Summary; SU = sulfonylurea.

Table S1 3 Fully Specified Hypoglycemia Model 1: SF-12 PCS

| Variable (reference) | Regression Coefficient | Standard Error | P Value | 95% CI | |
| --- | --- | --- | --- | --- | --- |
|  |  |  |  | Lower | Upper |
| Hypoglycemia | −0.974 | 0.840 | 0.247 | −2.623 | 0.675 |
| Age >65 years | −0.860 | 1.245 | 0.490 | −3.304 | 1.585 |
| Sex (male) | 0.509 | 0.803 | 0.527 | −1.069 | 2.086 |
| DM family history | 0.225 | 0.889 | 0.801 | −1.520 | 1.970 |
| Cohort: SU without insulin |  |  |  |  |  |
| AD with insulin | 0.468 | 0.934 | 0.616 | −1.366 | 2.302 |
| Non-SU AD without insulin | 1.488 | 0.944 | 0.116 | −0.366 | 3.341 |
| BMI | −0.355 | 0.051 | <.0001 | −0.456 | −0.255 |
| Duration of current AD medication | −0.103 | 0.309 | 0.740 | −0.710 | 0.505 |
| White | −0.047 | 0.887 | 0.958 | −1.789 | 1.694 |
| Hispanic or Latino | 0.035 | 1.384 | 0.980 | −2.683 | 2.752 |
| Marital status (currently not married) | −1.266 | 0.848 | 0.136 | −2.932 | 0.399 |
| Household income level | 1.546 | 0.306 | <.0001 | 0.945 | 2.147 |
| Education (lower than high school) | 5.346 | 2.312 | 0.021 | 0.805 | 9.886 |
| Region (Northeast) |  |  |  |  |  |
| Midwest | −0.756 | 1.829 | 0.680 | −4.348 | 2.836 |
| South | −1.028 | 1.515 | 0.498 | −4.002 | 1.947 |
| West | −1.940 | 2.059 | 0.346 | −5.983 | 2.102 |
| Diabetes duration (years) | −0.242 | 0.053 | <.0001 | −0.346 | −0.138 |
| Morisky adherence | −0.422 | 0.393 | 0.284 | −1.193 | 0.350 |

AD = antidiabetic drug; BMI = body mass index; DM = diabetes mellitus; SF-12 PCS = 12-item Short Form Health Survey Physical Component Summary; SU = sulfonylurea.

Table S1.4 Fully Specified Hypoglycemia Model 2: EQ-5D (US)

| Variable (reference) | Regression Coefficient | Standard Error | P Value | 95% CI | |
| --- | --- | --- | --- | --- | --- |
|  |  |  |  | Lower | Upper |
| Hypoglycemia | −0.014 | 0.014 | 0.303 | −0.04 | 0.013 |
| Hypoglycemia fear | −0.003 | 0.001 | <.0001 | −0.003 | −0.002 |
| Age >65 years | 0.015 | 0.019 | 0.424 | −0.022 | 0.053 |
| Sex (male) | 0.014 | 0.012 | 0.243 | −0.01 | 0.039 |
| DM family history | 0.001 | 0.014 | 0.991 | −0.027 | 0.027 |
| Cohort: SU without insulin |  |  |  |  |  |
| AD with insulin | −0.007 | 0.014 | 0.602 | −0.036 | 0.021 |
| Non-SU AD without insulin | −0.004 | 0.015 | 0.762 | −0.033 | 0.024 |
| BMI | −0.004 | 0.001 | <.0001 | −0.005 | −0.002 |
| Duration of current AD medication | 0.001 | 0.005 | 0.94 | −0.010 | 0.009 |
| White | −0.021 | 0.014 | 0.124 | −0.048 | 0.006 |
| Hispanic or Latino | 0.016 | 0.021 | 0.45 | −0.026 | 0.058 |
| Marital status (currently not married) | −0.012 | 0.013 | 0.351 | −0.038 | 0.013 |
| Household income level | 0.01 | 0.005 | 0.028 | 0.001 | 0.02 |
| Education (lower than high school) | 0.096 | 0.035 | 0.007 | 0.026 | 0.165 |
| Region (Northeast) |  |  |  |  |  |
| Midwest | −0.013 | 0.028 | 0.656 | −0.068 | 0.043 |
| South | −0.011 | 0.023 | 0.623 | −0.057 | 0.034 |
| West | 0.004 | 0.032 | 0.911 | −0.058 | 0.066 |
| Diabetes duration (years) | −0.002 | 0.001 | 0.029 | −0.003 | 0.001 |
| Morisky adherence | −0.006 | 0.006 | 0.33 | −0.018 | 0.006 |

AD = antidiabetic drug; BMI = body mass index; DM = diabetes mellitus; EQ-5D = EuroQol-5D index; SU = sulfonylurea.

Table S1.5 Fully Specified Hypoglycemia Model 2: SF-12 MCS

| Variable (reference) | Regression Coefficient | Standard Error | P Value | 95% CI | |
| --- | --- | --- | --- | --- | --- |
|  |  |  |  | Lower | Upper |
| Hypoglycemia | −1.937 | 0.842 | 0.022 | −3.591 | −0.283 |
| Hypoglycemia fear | −0.162 | 0.024 | <.0001 | −0.21 | −0.115 |
| Age >65 years | 2.171 | 1.191 | 0.069 | −0.168 | 4.511 |
| Sex (male) | 1.53 | 0.77 | 0.047 | 0.018 | 3.041 |
| DM family history | −0.404 | 0.851 | 0.635 | −2.074 | 1.266 |
| Cohort: SU without insulin |  |  |  |  |  |
| AD with insulin | −0.316 | 0.895 | 0.724 | −2.074 | 1.441 |
| Non-SU AD without insulin | −1.126 | 0.905 | 0.214 | −2.903 | 0.651 |
| BMI | −0.087 | 0.049 | 0.079 | −0.183 | 0.01 |
| Duration of current AD medication | 0.182 | 0.297 | 0.54 | −0.4 | 0.764 |
| White | −1.521 | 0.856 | 0.076 | −3.201 | 0.159 |
| Hispanic or Latino | −1.273 | 1.329 | 0.339 | −3.883 | 1.337 |
| Marital status (currently not married) | 0.705 | 0.812 | 0.385 | −0.888 | 2.299 |
| Household income level | −0.05 | 0.297 | 0.866 | −0.633 | 0.533 |
| Education (lower than high school) | 5.124 | 2.213 | 0.021 | 0.779 | 9.469 |
| Region (Northeast) |  |  |  |  |  |
| Midwest | 1.33 | 1.751 | 0.448 | −2.108 | 4.768 |
| South | 0.504 | 1.45 | 0.728 | −2.343 | 3.351 |
| West | 2.653 | 1.97 | 0.179 | −1.216 | 6.521 |
| Diabetes duration (years) | 0.043 | 0.051 | 0.4 | −0.058 | 0.144 |
| Morisky adherence | −1.899 | 0.378 | <.0001 | −2.642 | −1.155 |

AD = antidiabetic drug; BMI = body mass index; DM = diabetes mellitus; SF-12 MCS = 12-item Short Form Health Survey Mental Component Summary; SU = sulfonylurea.

Table S1.6 Fully Specified Hypoglycemia Model 2: SF-12 PCS

| Variable (reference) | Regression Coefficient | Standard Error | P Value | 95% CI | |
| --- | --- | --- | --- | --- | --- |
|  |  |  |  | Lower | Upper |
| Hypoglycemia | 0.579 | 0.857 | 0.500 | −1.104 | 2.262 |
| Hypoglycemia fear | −0.149 | 0.025 | <.0001 | −0.197 | −0.101 |
| Age >65 years | −1.034 | 1.212 | 0.394 | −3.414 | 1.347 |
| Sex (male) | 0.258 | 0.783 | 0.742 | −1.280 | 1.796 |
| DM family history | 0.133 | 0.866 | 0.878 | −1.567 | 1.832 |
| Cohort: SU without insulin |  |  |  |  |  |
| AD with insulin | 0.757 | 0.911 | 0.406 | −1.032 | 2.546 |
| Non-SU AD without insulin | 1.138 | 0.921 | 0.217 | −0.670 | 2.946 |
| BMI | −0.370 | 0.050 | <.0001 | −0.469 | −0.272 |
| Duration of current AD medication | −0.208 | 0.302 | 0.491 | −0.800 | 0.385 |
| White | −0.721 | 0.871 | 0.408 | −2.431 | 0.989 |
| Hispanic or Latino | 0.742 | 1.353 | 0.583 | −1.914 | 3.398 |
| Marital status (currently not married) | −1.267 | 0.826 | 0.126 | −2.888 | 0.355 |
| Household income level | 1.249 | 0.302 | <.0001 | 0.656 | 1.842 |
| Education (lower than high school) | 5.472 | 2.252 | 0.015 | 1.050 | 9.894 |
| Region (Northeast) |  |  |  |  |  |
| Midwest | −0.530 | 1.782 | 0.766 | −4.029 | 2.969 |
| South | −0.806 | 1.475 | 0.585 | −3.703 | 2.091 |
| West | −1.982 | 2.005 | 0.323 | −5.919 | 1.954 |
| Diabetes duration (years) | −0.187 | 0.052 | 0.000 | −0.290 | −0.084 |
| Morisky adherence | −0.156 | 0.385 | 0.685 | −0.912 | 0.600 |

AD = antidiabetic drug; BMI = body mass index; DM = diabetes mellitus; SF-12 PCS = 12-item Short Form Health Survey Physical Component Summary; SU = sulfonylurea.

Table S2.0 Hypoglycemia Model 1: Insulin Subgroup Measured by EQ-5D(US)

| Variable (reference) | Regression Coefficient | Standard Error | P Value | 95% CI | |
| --- | --- | --- | --- | --- | --- |
|  |  |  |  | Lower | Upper |
| Hypoglycemia | −0.001 | 0.029 | 0.960 | −0.058 | 0.055 |
| Age >65 years | 0.036 | 0.033 | 0.281 | −0.029 | 0.101 |
| Sex (male) | 0.025 | 0.023 | 0.270 | −0.020 | 0.070 |
| DM family history | 0.046 | 0.028 | 0.107 | −0.010 | 0.102 |
| BMI | −0.003 | 0.001 | 0.027 | −0.006 | 0.000 |
| Duration of current AD medication | 0.004 | 0.008 | 0.614 | −0.012 | 0.020 |
| White | −0.028 | 0.026 | 0.282 | −0.078 | 0.023 |
| Hispanic or Latino | 0.015 | 0.041 | 0.719 | −0.065 | 0.095 |
| Marital status (currently not married) | −0.042 | 0.025 | 0.089 | −0.091 | 0.006 |
| Household income level | 0.027 | 0.009 | 0.003 | 0.010 | 0.044 |
| Education (lower than high school) | 0.148 | 0.070 | 0.036 | 0.010 | 0.285 |
| Region (Northeast) |  |  |  |  |  |
| Midwest | −0.001 | 0.050 | 0.987 | −0.100 | 0.098 |
| South | −0.052 | 0.040 | 0.189 | −0.130 | 0.026 |
| West | −0.057 | 0.062 | 0.361 | −0.179 | 0.065 |
| Diabetes duration (years) | −0.003 | 0.001 | 0.030 | −0.005 | 0.000 |
| Morisky adherence | −0.016 | 0.011 | 0.123 | −0.037 | 0.004 |

AD = antidiabetic drug; BMI = body mass index; DM = diabetes mellitus; EQ-5D = EuroQol-5D index.

Table S2.1 Hypoglycemia Model 1: Insulin Subgroup Measured by SF-12 MCS

| Variable (reference) | Regression Coefficient | Standard Error | P Value | 95% CI | |
| --- | --- | --- | --- | --- | --- |
|  |  |  |  | Lower | Upper |
| Hypoglycemia | −2.506 | 1.679 | 0.137 | −5.813 | 0.801 |
| Age >65 years | 3.699 | 1.935 | 0.057 | −0.112 | 7.511 |
| Sex (male) | 2.154 | 1.328 | 0.106 | −0.462 | 4.771 |
| DM family history | 1.170 | 1.671 | 0.485 | −2.122 | 4.461 |
| BMI | −0.092 | 0.085 | 0.279 | −0.259 | 0.075 |
| Duration of current AD medication | 0.277 | 0.484 | 0.567 | −0.677 | 1.232 |
| White | 0.081 | 1.498 | 0.957 | −2.871 | 3.033 |
| Hispanic or Latino | −2.239 | 2.380 | 0.348 | −6.929 | 2.450 |
| Marital status (currently not married) | 0.080 | 1.449 | 0.956 | −2.775 | 2.935 |
| Household income level | 0.802 | 0.519 | 0.123 | −0.220 | 1.823 |
| Education (lower than high school) | 7.987 | 4.105 | 0.053 | −0.101 | 16.075 |
| Region (Northeast) |  |  |  |  |  |
| Midwest | −0.425 | 2.945 | 0.885 | −6.226 | 5.377 |
| South | −2.761 | 2.326 | 0.237 | −7.344 | 1.822 |
| West | −3.979 | 3.639 | 0.275 | −11.148 | 3.189 |
| Diabetes duration (years) | −0.009 | 0.077 | 0.910 | −0.160 | 0.142 |
| Morisky adherence | −2.741 | 0.621 | <.0001 | −3.964 | −1.518 |

AD = antidiabetic drug; BMI = body mass index; DM = diabetes mellitus; SF-12 MCS = 12-item Short Form Health Survey Mental Component Summary.

Table S2.2 Hypoglycemia Model 1: Insulin Subgroup Measured by SF-12 PCS

| Variable (reference) | Regression Coefficient | Standard Error | P Value | 95% CI | |
| --- | --- | --- | --- | --- | --- |
|  |  |  |  | Lower | Upper |
| Hypoglycemia | −1.668 | 1.815 | 0.359 | −5.244 | 1.908 |
| Age >65 years | 0.176 | 2.092 | 0.933 | −3.946 | 4.298 |
| Sex (male) | 0.520 | 1.436 | 0.718 | −2.310 | 3.349 |
| DM family history | 2.192 | 1.807 | 0.226 | −1.367 | 5.752 |
| BMI | −0.351 | 0.092 | 0.000 | −0.531 | −0.170 |
| Duration of current AD medication | −0.301 | 0.524 | 0.566 | −1.333 | 0.731 |
| White | −1.691 | 1.620 | 0.298 | −4.883 | 1.501 |
| Hispanic or Latino | 0.921 | 2.574 | 0.721 | −4.150 | 5.992 |
| Marital status (currently not married) | −3.687 | 1.567 | 0.020 | −6.775 | −0.599 |
| Household income level | 1.860 | 0.561 | 0.001 | 0.755 | 2.965 |
| Education (lower than high school) | 6.591 | 4.439 | 0.139 | −2.155 | 15.338 |
| Region (Northeast) |  |  |  |  |  |
| Midwest | −0.048 | 3.185 | 0.988 | −6.321 | 6.226 |
| South | −3.373 | 2.516 | 0.181 | −8.329 | 1.583 |
| West | −3.515 | 3.935 | 0.373 | −11.267 | 4.237 |
| Diabetes duration (years) | −0.288 | 0.083 | 0.001 | −0.451 | −0.125 |
| Morisky adherence | 0.046 | 0.671 | 0.945 | −1.276 | 1.368 |

AD = antidiabetic drug; BMI = body mass index; DM = diabetes mellitus; SF-12 PCS = 12-item Short Form Health Survey Physical Component Summary.

Table S2.3 Hypoglycemia Model 2: Insulin Subgroup Measured by EQ-5D(US)

| Variable (reference) | Regression Coefficient | Standard Error | P Value | 95% CI | |
| --- | --- | --- | --- | --- | --- |
|  |  |  |  | Lower | Upper |
| Hypoglycemia | 0.029 | 0.029 | 0.311 | −0.027 | 0.086 |
| Hypoglycemia fear | −0.003 | 0.001 | <.0001 | −0.004 | −0.001 |
| Age >65 years | 0.034 | 0.032 | 0.281 | −0.028 | 0.097 |
| Sex (male) | 0.022 | 0.022 | 0.325 | −0.022 | 0.065 |
| DM family history | 0.036 | 0.028 | 0.197 | −0.019 | 0.090 |
| BMI | −0.004 | 0.001 | 0.014 | −0.006 | −0.001 |
| Duration of current AD medication | 0.003 | 0.008 | 0.707 | −0.013 | 0.019 |
| White | −0.039 | 0.025 | 0.118 | −0.088 | 0.010 |
| Hispanic or Latino | 0.038 | 0.040 | 0.336 | −0.040 | 0.116 |
| Marital status (currently not married) | −0.040 | 0.024 | 0.092 | −0.088 | 0.007 |
| Household income level | 0.020 | 0.009 | 0.024 | 0.003 | 0.037 |
| Education (lower than high school) | 0.175 | 0.068 | 0.011 | 0.041 | 0.309 |
| Region (Northeast) |  |  |  |  |  |
| Midwest | 0.006 | 0.049 | 0.904 | −0.090 | 0.102 |
| South | −0.042 | 0.038 | 0.277 | −0.118 | 0.034 |
| West | −0.053 | 0.060 | 0.374 | −0.172 | 0.065 |
| Diabetes duration (years) | −0.002 | 0.001 | 0.205 | −0.004 | 0.001 |
| Morisky adherence | −0.012 | 0.010 | 0.251 | −0.032 | 0.008 |

AD = antidiabetic drug; BMI = body mass index; DM = diabetes mellitus; EQ-5D = EuroQol-5D index.

Table S2.4 Hypoglycemia Model 2: Insulin Subgroup Measured by SF-12 MCS

| Variable (reference) | Regression Coefficient | Standard Error | P Value | 95% CI | |
| --- | --- | --- | --- | --- | --- |
|  |  |  |  | Lower | Upper |
| Hypoglycemia | −0.285 | 1.649 | 0.863 | −3.534 | 2.965 |
| Hypoglycemia fear | −0.188 | 0.036 | <.0001 | −0.259 | −0.116 |
| Age >65 years | 3.613 | 1.836 | 0.050 | −0.005 | 7.230 |
| Sex (male) | 1.905 | 1.261 | 0.132 | −0.579 | 4.390 |
| DM family history | 0.428 | 1.592 | 0.788 | −2.708 | 3.564 |
| BMI | −0.111 | 0.081 | 0.168 | −0.270 | 0.047 |
| Duration of current anti-diabetic medication | 0.193 | 0.460 | 0.675 | −0.713 | 1.099 |
| White | −0.756 | 1.431 | 0.598 | −3.575 | 2.063 |
| Hispanic or Latino | −0.520 | 2.283 | 0.820 | −5.017 | 3.978 |
| Marital status (currently not married) | 0.209 | 1.375 | 0.879 | −2.501 | 2.919 |
| Household income level | 0.283 | 0.502 | 0.573 | −0.706 | 1.273 |
| Education (lower than high school) | 9.970 | 3.914 | 0.012 | 2.258 | 17.681 |
| Region (Northeast) |  |  |  |  |  |
| Midwest | 0.061 | 2.796 | 0.983 | −5.447 | 5.570 |
| South | −2.013 | 2.212 | 0.364 | −6.371 | 2.345 |
| West | −3.735 | 3.453 | 0.281 | −10.538 | 3.068 |
| Diabetes duration (years) | 0.078 | 0.075 | 0.294 | −0.069 | 0.225 |
| Morisky adherence | −2.412 | 0.592 | <.0001 | −3.579 | −1.245 |

AD = antidiabetic drug; BMI = body mass index; DM = diabetes mellitus; SF-12 MCS = 12-item Short Form Health Survey Mental Component Summary.

Table S2.5 Hypoglycemia Model 2: Insulin Subgroup Measured by SF-12 PCS

| Variable (reference) | Regression Coefficient | Standard Error | P Value | 95% CI | |
| --- | --- | --- | --- | --- | --- |
|  |  |  |  | Lower | Upper |
| Hypoglycemia | −0.358 | 1.854 | 0.847 | −4.011 | 3.296 |
| Hypoglycemia fear | −0.111 | 0.041 | 0.007 | −0.191 | −0.031 |
| Age >65 years | 0.125 | 2.064 | 0.952 | −3.942 | 4.192 |
| Sex (Male) | 0.373 | 1.418 | 0.793 | −2.421 | 3.167 |
| DM family history | 1.755 | 1.790 | 0.328 | −1.771 | 5.281 |
| BMI | −0.362 | 0.091 | <.0001 | −0.541 | −0.184 |
| Duration of current AD medication | −0.351 | 0.517 | 0.498 | −1.370 | 0.668 |
| White | −2.185 | 1.609 | 0.176 | −5.355 | 0.985 |
| Hispanic or Latino | 1.936 | 2.567 | 0.452 | −3.121 | 6.992 |
| Marital status (currently not married) | −3.611 | 1.547 | 0.020 | −6.658 | −0.564 |
| Household income level | 1.554 | 0.565 | 0.006 | 0.442 | 2.666 |
| Education (lower than high school) | 7.761 | 4.401 | 0.079 | −0.910 | 16.431 |
| Region (Northeast) |  |  |  |  |  |
| Midwest | 0.239 | 3.144 | 0.939 | −5.954 | 6.432 |
| South | −2.932 | 2.487 | 0.240 | −7.832 | 1.968 |
| West | −3.371 | 3.882 | 0.386 | −11.020 | 4.278 |
| Diabetes duration (years) | −0.237 | 0.084 | 0.005 | −0.402 | −0.072 |
| Morisky adherence | 0.240 | 0.666 | 0.719 | −1.072 | 1.552 |

AD = antidiabetic drug; BMI = body mass index; DM = diabetes mellitus; SF-12 PCS = 12-item Short Form Health Survey Physical Component Summary.

Table S3.0 Hypoglycemia Model 1: Sulfonylurea Subgroup Measured by EQ-5D (US)

| Variable (reference) | Regression Coefficient | Standard Error | P Value | 95% CI | |
| --- | --- | --- | --- | --- | --- |
|  |  |  |  | Lower | Upper |
| Hypoglycemia | −0.077 | 0.023 | 0.001 | −0.123 | −0.031 |
| Age >65 years | −0.032 | 0.040 | 0.428 | −0.110 | 0.047 |
| Sex(male) | 0.004 | 0.024 | 0.861 | −0.044 | 0.052 |
| DM family history | −0.032 | 0.024 | 0.176 | −0.079 | 0.015 |
| BMI | −0.005 | 0.001 | 0.000 | −0.008 | −0.002 |
| Duration of current AD medication | 0.005 | 0.009 | 0.535 | −0.012 | 0.022 |
| White | 0.040 | 0.027 | 0.144 | −0.014 | 0.094 |
| Hispanic or Latino | −0.061 | 0.039 | 0.125 | −0.138 | 0.017 |
| Marital status (currently not married) | 0.006 | 0.023 | 0.805 | −0.041 | 0.052 |
| Household income level | −0.003 | 0.009 | 0.754 | −0.020 | 0.015 |
| Education (lower than high school) | −0.030 | 0.066 | 0.650 | −0.160 | 0.100 |
| Region (Northeast) |  |  |  |  |  |
| Midwest | 0.033 | 0.048 | 0.495 | −0.062 | 0.127 |
| South | 0.035 | 0.041 | 0.399 | −0.046 | 0.115 |
| West | 0.010 | 0.061 | 0.866 | −0.111 | 0.131 |
| Diabetes duration (years) | −0.001 | 0.002 | 0.358 | −0.004 | 0.002 |
| Morisky adherence | −0.008 | 0.013 | 0.526 | −0.033 | 0.017 |

AD = antidiabetic drug; BMI = body mass index; DM = diabetes mellitus; EQ-5D = EuroQol-5D index.

Table S3.1 Hypoglycemia Model 1: Sulfonylurea Subgroup Measured by SF-12 MCS

| Variable (reference) | Regression Coefficient | Standard Error | P Value | 95% CI | |
| --- | --- | --- | --- | --- | --- |
|  |  |  |  | Lower | Upper |
| Hypoglycemia | −6.531 | 1.558 | <.0001 | −9.606 | −3.456 |
| Age >65 years | 0.049 | 2.655 | 0.985 | −5.191 | 5.290 |
| Sex (male) | 1.791 | 1.627 | 0.272 | −1.419 | 5.002 |
| DM family history | −0.080 | 1.597 | 0.960 | −3.233 | 3.072 |
| BMI | −0.039 | 0.091 | 0.671 | −0.217 | 0.140 |
| Duration of current AD medication | 0.594 | 0.570 | 0.299 | −0.531 | 1.719 |
| White | 0.053 | 1.829 | 0.977 | −3.556 | 3.661 |
| Hispanic or Latino | −4.689 | 2.638 | 0.077 | −9.895 | 0.518 |
| Marital status (currently not married) | −0.695 | 1.573 | 0.659 | −3.798 | 2.409 |
| Household income level | −0.509 | 0.595 | 0.394 | −1.683 | 0.665 |
| Education (low than high school) | 1.678 | 4.427 | 0.705 | −7.059 | 10.416 |
| Region (Northeast) |  |  |  |  |  |
| Midwest | 3.994 | 3.210 | 0.215 | −2.341 | 10.330 |
| South | 4.599 | 2.735 | 0.095 | −0.800 | 9.997 |
| West | 8.199 | 4.106 | 0.047 | 0.096 | 16.302 |
| Diabetes duration (years) | −0.036 | 0.101 | 0.725 | −0.234 | 0.163 |
| Morisky adherence | −1.402 | 0.850 | 0.101 | −3.080 | 0.277 |

AD = antidiabetic drug; BMI = body mass index; DM = diabetes mellitus; SF-12 MCS = 12-item Short Form Health Survey Mental Component Summary.

Table S3.2 Hypoglycemia Model 1: Sulfonylurea Subgroup Measured by SF-12 PCS

| Variable (reference) | Regression Coefficient | Standard Error | P Value | 95% CI | |
| --- | --- | --- | --- | --- | --- |
|  |  |  |  | Lower | Upper |
| Hypoglycemia | −1.815 | 1.541 | 0.241 | −4.857 | 1.226 |
| Age >65 years | −3.623 | 2.627 | 0.170 | −8.807 | 1.561 |
| Sex (male) | −2.519 | 1.609 | 0.119 | −5.695 | 0.656 |
| DM family history | −1.998 | 1.580 | 0.208 | −5.116 | 1.121 |
| BMI | −0.514 | 0.090 | <.0001 | −0.691 | −0.338 |
| Duration of current anti-diabetic medication | 0.794 | 0.564 | 0.161 | −0.319 | 1.906 |
| White | 2.779 | 1.809 | 0.126 | −0.791 | 6.348 |
| Hispanic or Latino | 1.020 | 2.609 | 0.696 | −4.129 | 6.170 |
| Marital status (currently not married) | 0.102 | 1.556 | 0.948 | −2.968 | 3.172 |
| Household income level | 1.515 | 0.588 | 0.011 | 0.354 | 2.677 |
| Education (lower than high school) | 0.669 | 4.379 | 0.879 | −7.974 | 9.311 |
| Region (Northeast) |  |  |  |  |  |
| Midwest | 0.320 | 3.175 | 0.920 | −5.947 | 6.587 |
| South | 1.566 | 2.706 | 0.564 | −3.774 | 6.906 |
| West | −1.474 | 4.061 | 0.717 | −9.489 | 6.541 |
| Diabetes duration (years) | −0.146 | 0.100 | 0.146 | −0.342 | 0.051 |
| Morisky adherence | −1.037 | 0.841 | 0.219 | −2.697 | 0.623 |

AD = antidiabetic drug; BMI = body mass index; DM = diabetes mellitus; SF-12 PCS = 12-item Short Form Health Survey Physical Component Summary.

Table S3.3 Hypoglycemia Model 2: Sulfonylurea Subgroup Measured by EQ-5D (US)

| Variable (reference) | Regression Coefficient | Standard Error | P Value | 95% CI | |
| --- | --- | --- | --- | --- | --- |
|  |  |  |  | Lower | Upper |
| Hypoglycemia | −0.060 | 0.024 | 0.014 | −0.107 | −0.013 |
| Hypoglycemia Fear | −0.002 | 0.001 | 0.014 | −0.004 | 0.000 |
| Age >65 years | −0.041 | 0.039 | 0.298 | −0.118 | 0.036 |
| Sex (male) | −0.007 | 0.024 | 0.787 | −0.055 | 0.041 |
| DM family history | −0.032 | 0.023 | 0.181 | −0.078 | 0.015 |
| BMI | −0.005 | 0.001 | 0.000 | −0.008 | −0.003 |
| Duration of current AD medication | 0.003 | 0.008 | 0.716 | −0.014 | 0.020 |
| White | 0.034 | 0.027 | 0.204 | −0.019 | 0.088 |
| Hispanic or Latino | −0.051 | 0.039 | 0.192 | −0.128 | 0.026 |
| Marital status (currently not married) | 0.012 | 0.023 | 0.604 | −0.034 | 0.058 |
| Household income level | −0.005 | 0.009 | 0.600 | −0.022 | 0.013 |
| Education (lower than high school) | −0.055 | 0.066 | 0.402 | −0.185 | 0.075 |
| Region (Northeast) |  |  |  |  |  |
| Midwest | 0.028 | 0.047 | 0.548 | −0.065 | 0.122 |
| South | 0.029 | 0.040 | 0.466 | −0.050 | 0.109 |
| West | 0.018 | 0.060 | 0.765 | −0.101 | 0.137 |
| Diabetes duration (years) | −0.001 | 0.001 | 0.487 | −0.004 | 0.002 |
| Morisky adherence | −0.002 | 0.013 | 0.876 | −0.027 | 0.023 |

AD = antidiabetic drug; BMI = body mass index; DM = diabetes mellitus; EQ-5D = EuroQol-5D index.

Table S3.4 Hypoglycemia Model 2: Sulfonylurea Subgroup Measured by SF-12 MCS

| Variable (reference) | Regression Coefficient | Standard Error | P Value | 95% CI | |
| --- | --- | --- | --- | --- | --- |
|  |  |  |  | Lower | Upper |
| Hypoglycemia | −5.300 | 1.597 | 0.001 | −8.453 | −2.147 |
| Hypoglycemia fear | −0.140 | 0.052 | 0.008 | −0.243 | −0.038 |
| Age >65 years | −0.645 | 2.622 | 0.806 | −5.819 | 4.530 |
| Sex (male) | 1.004 | 1.625 | 0.537 | −2.203 | 4.211 |
| DM family history | −0.023 | 1.570 | 0.989 | −3.121 | 3.076 |
| BMI | −0.056 | 0.089 | 0.532 | −0.232 | 0.120 |
| Duration of current AD medication | 0.433 | 0.563 | 0.443 | −0.679 | 1.545 |
| White | −0.356 | 1.803 | 0.844 | −3.915 | 3.202 |
| Hispanic or Latino | −3.987 | 2.605 | 0.128 | −9.128 | 1.154 |
| Marital status (currently not married) | −0.237 | 1.555 | 0.879 | −3.305 | 2.832 |
| Household income level | −0.642 | 0.587 | 0.276 | −1.799 | 0.516 |
| Education (lower than high school) | −0.165 | 4.403 | 0.970 | −8.856 | 8.526 |
| Region (Northeast) |  |  |  |  |  |
| Midwest | 3.683 | 3.156 | 0.245 | −2.547 | 9.912 |
| South | 4.225 | 2.691 | 0.118 | −1.086 | 9.537 |
| West | 8.765 | 4.040 | 0.031 | 0.793 | 16.738 |
| Diabetes duration (years) | −0.010 | 0.099 | 0.918 | −0.207 | 0.186 |
| Morisky adherence | −0.961 | 0.851 | 0.261 | −2.641 | 0.720 |

AD = antidiabetic drug; BMI = body mass index; DM = diabetes mellitus; SF-12 MCS = 12-item Short Form Health Survey Mental Component Summary.

Table S3.5 Hypoglycemia Model 2: Sulfonylurea Subgroup Measured by SF-12 PCS

| Variable (reference) | Regression Coefficient | Standard Error | P Value | 95% CI | |
| --- | --- | --- | --- | --- | --- |
|  |  |  |  | Lower | Upper |
| Hypoglycemia | 0.056 | 1.535 | 0.971 | −2.973 | 3.084 |
| Hypoglycemia fear | −0.213 | 0.050 | <.0001 | −0.311 | −0.114 |
| Age >65 years | −4.679 | 2.518 | 0.065 | −9.649 | 0.292 |
| Sex (male) | −3.716 | 1.561 | 0.018 | −6.796 | −0.636 |
| DM family history | −1.910 | 1.508 | 0.207 | −4.886 | 1.066 |
| BMI | −0.541 | 0.086 | <.0001 | −0.710 | −0.372 |
| Duration of current AD medication | 0.549 | 0.541 | 0.312 | −0.519 | 1.617 |
| White | 2.158 | 1.732 | 0.215 | −1.261 | 5.576 |
| Hispanic or Latino | 2.086 | 2.502 | 0.406 | −2.853 | 7.024 |
| Marital status (currently not married) | 0.798 | 1.493 | 0.594 | −2.149 | 3.745 |
| Household income level | 1.313 | 0.563 | 0.021 | 0.201 | 2.426 |
| Education (low than high school) | −2.133 | 4.230 | 0.615 | −10.482 | 6.215 |
| Region (Northeast) |  |  |  |  |  |
| Midwest | −0.154 | 3.032 | 0.960 | −6.138 | 5.830 |
| South | 0.998 | 2.585 | 0.700 | −4.104 | 6.100 |
| West | −0.613 | 3.880 | 0.875 | −8.272 | 7.046 |
| Diabetes duration (years) | −0.107 | 0.096 | 0.263 | −0.296 | 0.081 |
| Morisky adherence | −0.367 | 0.818 | 0.654 | −1.981 | 1.247 |

AD = antidiabetic drug; BMI = body mass index; DM = diabetes mellitus; SF-12 PCS = 12-item Short Form Health Survey Physical Component Summary.
